# The impact of anterior knee displacement on knee joint load during the forward bow step in Tai Chi

**DOI:** 10.3389/fbioe.2024.1458737

**Published:** 2024-10-15

**Authors:** Lijun Hua, Gengchao Bi, Yanlong Zhang, Kai Wang, Jiao Liu

**Affiliations:** ^1^ College of Physical Education and Training, Harbin Sport University, Harbin, China; ^2^ Graduate school, Harbin Sport University, Harbin, China; ^3^ College of Sports and Health Sciences, Mudanjiang Normal University, Mudanjiang, China

**Keywords:** Tai Chi, anterior knee displacement, forward bow step, knee joint load, muscle force

## Abstract

**Background:**

While the forward bow step is a crucial component of Tai Chi (TC) practice, little research has been conducted on its impact on knee joint load and muscle coordination. This study aims to investigate the effects of three different knee forward positions during the TC forward bow step on knee joint loading.

**Methods:**

Twenty TC practitioners were recruited, and motion capture systems, force platforms, and surface electromyography were utilized to synchronously collect biomechanical parameters of three types of forward bow steps: knee joint not exceeding the tip of the foot (NETT), knee joint forward movement level with the tip of the foot (LTT), and knee joint forward movement exceeding the tip of the foot (ETT). Ligament and muscle forces were calculated using OpenSim software for musculoskeletal modeling and simulation. One-way ANOVA was used to analyze the variations of the indicators during the peak anterior displacement of the knee joint in three movements. Additionally, spm1d one-way ANOVA was employed to examine the variations in the one-dimensional curve of the indicators throughout the entire movement process.

**Results:**

Compared with LTT and ETT, the NETT posture was associated with significantly decreased knee flexion angle (F = 27.445, *p* = 0.001), knee anterior-posterior translation (F = 36.07, *p* < 0.001), flexion-extension torque (F = 22.232, *p* = 0.001), ligament force (F = 9.055, *p* = 0.011). Additionally, there was also a significant reduction in muscle strength, including quadriceps (F = 62.9, *p* < 0.001), long biceps femoris (F = 18.631, *p* = 0.002), lateral gastrocnemius (F = 24.933, *p* = 0.001) and soleus (F = 7.637, *p* = 0.017).

**Conclusion:**

This study further confirms that in the forward lunge movement of Tai Chi, the knee joint load is mainly concentrated during the forward movement phase. Compared to the knee joint load at the NETT position, the load is greater at the LTT position; and compared to the LTT position, the load is even greater at the ETT position.

## 1 Introduction

Tai Chi (TC) is widely recognized as an effective method for promoting and maintaining health, particularly among middle-aged and older adults. Recent studies have shown that it can significantly improve balance, flexibility, and muscular coordination (Wayne et al., 2021; Wehner et al., 2021; Yang et al., 2022). Due to its low intensity and slow pace, TC is especially suitable for middle-aged and elderly individuals. However, certain movements can still exert considerable pressure on the knee joints (Wang et al., 2019; Law and Li, 2022).

Knee joint health issues are widespread in sports activities, significantly impacting individuals’ mobility and quality of life (Lam and Markbreiter, 2019). Research has identified a correlation between anterior cruciate ligament (ACL) injuries and anterior knee displacement (AKD) during movement (Mitchell et al., 2021). In sports such as basketball and skiing, athletes often extend their knee joints past the tip of their toes when making rapid stops, turns, or downhill slides, thereby increasing the risk of ACL injuries (Leppanen et al., 2021; Ruedl et al., 2023).

In TC movements, the forward bow step occurs most frequently, which is an important exercise of the lower limb and involves many repetitive movements characterized by shifting weight forward or backward. During this process, the knee joint also moves forward and backward repetitively (Wang C. et al., 2022). Anterior Knee Displacement (AKD) describes the forward movement of the knee during motion, and there are three types, knee not exceeding the tip of the foot (NETT), knee aligning with the tip of the foot (LTT), and knee exceeding the tip of the foot (ETT) (Kernozek et al., 2018). TC masters usually advise practitioners to limit the forward movement of the front knee to avoid exceeding the tip of the toe, thereby reducing knee joint pressure and potential injury risks. However, the underlying biomechanical mechanisms require further investigation.

Given the pivotal role of the knee joint in skilled and repetitive activities like TC, investigating its biomechanical behavior within these practices is crucial for injury prevention (Wang et al., 2016). Furthermore, TC stepping techniques can lead to high peak knee extension moments (Wen et al., 2018), which may be associated with muscle imbalances, particularly between the medial and lateral quadriceps and between the semitendinosus and biceps femoris muscles (Liu et al., 2023).However, there is still a lack of extensive research on the specific biomechanical loads on the knee joint in TC, especially those that extend beyond the toe tip and their long-term implications for knee health. This necessitates further investigation to ascertain their impact. Musculoskeletal modeling methods enable a non-invasive evaluation of knee joint loading during motion, quantitatively calculating muscle contraction forces. This approach is vital for understanding musculoskeletal structure, joint pathology, and motor control strategies of the central nervous system, playing an important role in comprehending the stress placed on the knee joint structure during TC (Schellenberg et al., 2018; Bennett et al., 2022; Pang et al., 2024).

This study employed experimental methods to quantitatively analyze the specific effects of different anterior knee joint positions on knee angle, torque, the stress of anterior cruciate ligament, and surrounding muscles. The motor characteristics and loads of the knee joint were accurately measured and evaluated using motion capture techniques and musculoskeletal modeling. The study hypothesizes that an increased forward position of the front knee will lead to a rise in the mechanical load on the knee. This study aims to provide scientific training guidance for TC practitioners and offer new perspectives and data support for the field of exercise biomechanics.

## 2 Methods

### 2.1 Participants

Sample size estimation was conducted using GPower3.1.9.7, with a significance level (α) of 0.05 and a statistical power of 80%. The analysis determined that the minimum required sample size was thirteen participants. We recruited 20 TC practitioners, each with 3–5 years of experience and practicing more than five times per week. The participants had an average age of 35.5 ± 7.6 years, an average height of 173.5 ± 5.3 cm, and an average weight of 75.8 ± 4.5 kg. They had no history of lower limb injuries in the past year. The recruitment period for the participants was from October 23 to 5 November 2023. Before the experiment began, all participants were informed of the study procedures and content, and signed an informed consent form before getting involved in this study. This study received approval from the Ethics Committee of Harbin Sports University, with approval number 2024012.

### 2.2 Experimental equipment and procedures

The study utilized eight 600 series high-speed cameras (200Hz, Qualisys, Sweden) two 40 cm × 60 cm multi-axis biomechanics force plate (1,000 Hz, model, BP4000600; AMTI, United States) and A 16-channel Trigno^®^ wireless surface electromyography (EMG) system (2,000 Hz, DELSYS, United States). These devices collectively captured the subjects’ three-dimensional kinematics, dynamics, and surface EMG signals. Based on the OpenSim gait2392 Marker Set, as shown in Table 1, reflective markers were attached to the subjects’ bodies or clothing. Surface EMG sensors were placed on the subjects’ left rectus femoris, left vastus medialis, left vastus lateralis, left tibialis anterior, left medial gastrocnemius, right rectus femoris, right vastus medialis, right vastus lateralis, right tibialis anterior, and right medial gastrocnemius.

**TABLE 1:** Specific locations of the markers placed on the body.

Specific locations of the markers placed on the body		
Top of the head	Left/Right Anterior Superior Iliac Spine	Left/Right Lateral Ankle
Left/Right Temporal Region	Left/Right Upper Thigh	Left/Right Medial Ankle
Sternum	Left/Right Front of Thigh	Left/Right Heel
Left/Right Acromion	Left/Right Rear of Thigh	Left/Right Midfoot-Sup
Left/Right Biceps Brachii	Left/Right Lateral Knee	Left/Right Midfoot-Lat
Left/Right Elbow	Left/Right Medial Knee	Left/Right Toe Lateral
Left/Right Wrist Medial	Left/Right Shank of Upper	Left/Right Toe Medial
Left/Right Wrist Lateral	Left/Right Shank of Front	Left/Right Toe-Tip
Sacrum	Left/Right Shank of Rear	

**FIGURE 1:**
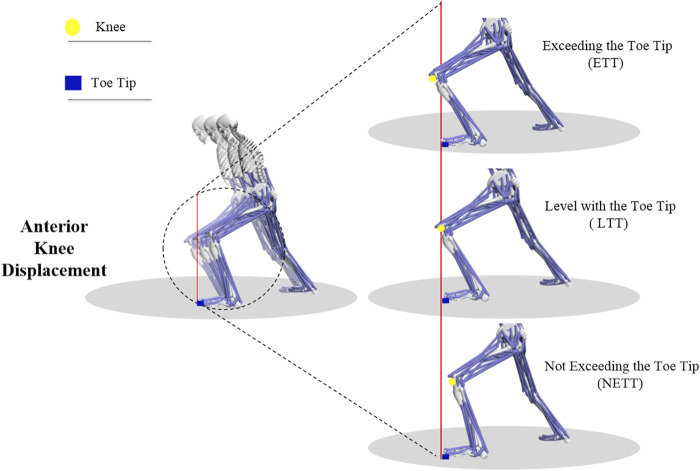
Schematic diagram of the actions for NETT, LTT, and ETT positions. A red line, vertical to the ground and originating from the tip of the foot, is used as the baseline in the figure. The yellow circle represents the knee, while the blue rectangle denotes the toe tip. This clearly illustrates the differences between ETT, LTT, and NETT.

Participants were instructed to perform the TC movement “Left Clasping the Sparrow’s Tail,” which involved executing three distinct positions of knee joint forward movement within the forward-stepping bow stance. These positions were defined based on the distance between the knee and the distal phalanx of the big toe. A line from the calcaneus to the distal end of the second metatarsal served as the baseline for reference. The distal phalanx of the big toe was used as the origin point, with a line perpendicular to the baseline forming a vertical plane. In this plane, the knee joint’s position relative to the distal phalanx of the big toe determined the classification of the movement positions: Not Exceeding the Toe Tip (NETT), Level with Toe Tip (LTT), and Exceeding the Toe Tip (ETT), as shown in Figure 1. In this study, we used a motion capture system to record the positions of the knee joint and toe. The quantification of knee advancement was achieved by calculating the relative positions of the knee joint and toe markers along the anterior-posterior direction. After performing statistical analysis on these displacement data, we calculated the average knee advancement distance and its standard deviation for participants in different postures. The results were as follows: NETT: −0.10 ± 0.03 m, LTT: −0.01 ± 0.04 m, ETT: 0.11 ± 0.01 m, where “−” indicates that the knee joint did not exceed the toe. The statistical difference among the three was significant (*p* < 0.01).

To ensure consistency and minimize variations in the body’s center of gravity during formal testing, participants were required to maintain a standardized posture. The action height was calculated using the formula: [(subject height × 0.89)−8.0]cm (Wen et al., 2018). This formula establishes a horizontal visual height reference, which helps control fluctuations in the center of gravity. The protocol was thoroughly practiced multiple times before the formal testing to guarantee uniformity.

The movement protocol required using a reference line orientation, formed by the connection between the center of the calcaneus and the distal end of the second metatarsal, and a knee joint movement direction with a horizontal plane projection of 0°. This method was adopted to eliminate the potential influence of footwork angle variations on the study. Participants were required to execute each movement successfully three times, with a 30-s interval between repetitions. The forward movement phase (FMP) and the backward movement phase (BMP) of the bow stance were determined by the data of vertical ground reaction force. It was described as FMP when the vertical ground reaction force exceeded 5N, and it was called BMP when the vertical ground reaction force was below 5N.

### 2.3 Musculoskeletal model

The basic model, modified by Xu et al. (2015), is derived from Simbody Gait2392 and includes a knee ligament model for purpose of simulation. (https://simtk.org/projects/kneeligament/). This model consists of 12 rigid bodies, 31 degrees of freedom (DOF), and 92 muscle actuators In particular, the knee joint in the model has three rotational degrees of freedom (flexion-extension, abduction-adduction, internal-external rotation) and three translational degrees of freedom (anterior-posterior, superior-inferior, medial-lateral). The knee ligaments are represented by ten elastic elements, which describe their geometric and mechanical properties.

### 2.4 Simulation process and data processing

Data processing was performed by OpenSim software. A 10 Hz low-pass Butterworth filter was applied to the raw kinetic data, whereas a 100 Hz low-pass Butterworth filter was used for the kinematic data (Sasaki and Neptune, 2010).

The entire OpenSim simulation process includes the following steps: First, the model and static kinematic data are scaled align with the body dimensions of the individual subjects, including height and limb length. To match the subjects’ body dimensions. This process enables us to recreate a scaled model that accurately reflects the geometric features of the actual individuals. Second, inverse kinematic operations are performed on the scaled model and dynamic kinematic data to obtain joint angle parameters. Third, the inverse kinematic results and force plate data are applied to the scaled model, followed by inverse dynamics to obtain joint torque data. Fourth, the residual reduction algorithm is used to optimize the kinematic and kinetic data. Finally, muscle control functions are calculated based on the optimized data to determine the strength of the muscles around the knee joint (Delp et al., 2007).

Surface electromyography (sEMG) signals were processed with a bandpass Butterworth filter in the range of 10–500 Hz and a 50 Hz notch filter. The data were rectified, the maximum value was normalized, and the root mean square (RMS) value was calculated (Ellenberger et al., 2021).The normalized sEMG signals were smoothed using a 50 ms sliding window with RMS and envelope functions to generate muscle activation curves, which were then used to validate the simulated muscle force results.

This study specifically focused on the changes in Knee’s three degrees of freedom, knee torque, the force changes of the anterior cruciate ligament (ACL) and the muscle activation patterns around knee joint. During the forward step of the “Left Grasping the Sparrow’s Tail” TC movement, all participants used their left leg as the front leg. The main indicators of the study included vertical ground reaction force (vGRF), knee anterior-posterior translation (KAPT), knee flexion and extension angle (KFA), knee abduction and adduction angle (KAA), and knee external and internal rotation angle (KRA); knee flexion and extension moment (KFM), knee abduction and adduction moment (KAM), and knee external and internal rotation moment (KRM); as well as the strength and activation patterns of key muscles, including the anterior cruciate ligament (ACL), rectus femoris (RF), vastus medialis (VM), vastus intermedius (VI), vastus lateralis (VL), biceps femoris long head (BFlh), biceps femoris short head (BFsh), tibialis posterior (TP), soleus, and gastrocnemius medialis (MG), and gastrocnemius lateralis (LG).

### 2.5 Data normalization and statistical analysis

The data for the forward bow step in TC were normalized over time, representing the action cycle from 0% to 100%. The knee torque and the strength of the muscles around the knee joint were normalized to body weight (BW). The average time at which the forward movement of the knee joint stops during the forward bow step cycle was marked by a dashed line on the time axis. A one-way analysis of variance (ANOVA) was used to evaluate differences in vertical ground reaction force, knee joint flexion angle, knee joint adduction angle, knee joint internal rotation angle, knee joint flexion torque, knee joint adduction torque, knee joint internal rotation torque, and ACL force among the NETT, LTT, and ETT groups. Before conducting ANOVA, normal distribution test and homogeneity of variance test were performed. When ANOVA results were statistically significant, multiple comparison tests were conducted: the Bonferroni method was used for homogeneous variance, and Tamhane’s T2 method was used for heterogeneous variance, with a significance level of 0.05. Additionally, the effect size η^2^ was calculated to comprehensively evaluate the practical significance of the findings. For the one-dimensional index curves of each group, the F-test SPM1d used in MATLAB 2021a was used for difference analysis, with a two-tailed test and a significance level of 0.05 (Pataky et al., 2016).

## 3 Results

**FIGURE 2:**
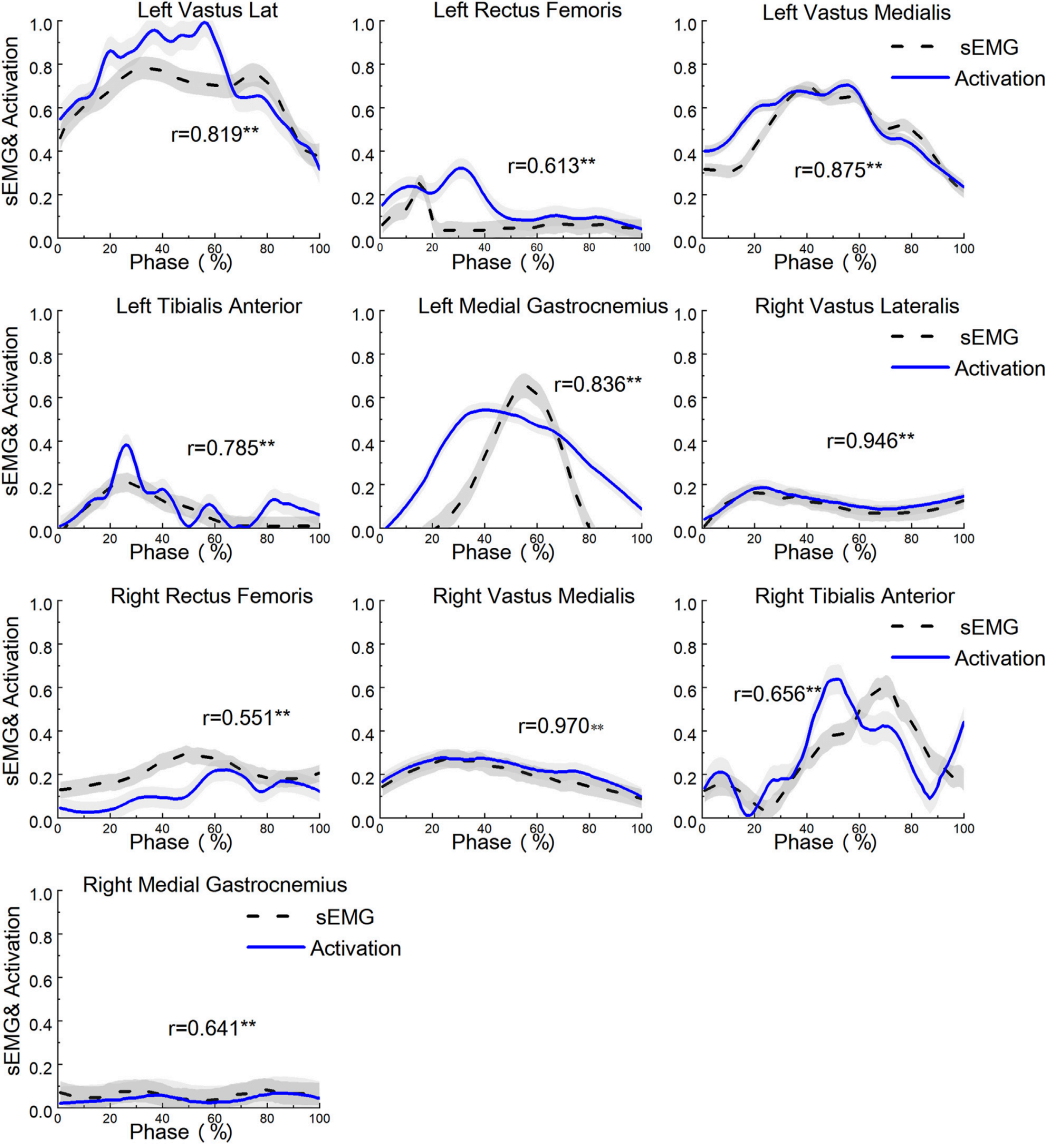
Model validation diagram. The range of activation values is from 0 (indicating complete inactivity) to 1 (indicating full activation). ** Indicates: p < 0.01.

In the model validation process, the accuracy and effectiveness of the model are evaluated by comparing the experimentally recorded surface electromyography (sEMG) signals with the simulated muscle activation values from the model. First, the sEMG signals undergo Root Mean Square (RMS) and envelope processing to calculate the muscle activation values (ranging from 0 to 1, representing no activation and full activation, respectively). Then, these activation values (sEMG) are compared and analyzed against the muscle activation values (Activation) generated by OpenSim. The results shown in Figure 2 indicate that the average Pearson correlation coefficient for the ten muscles is 0.76, demonstrating a significant positive correlation between the two. This verifies the reliability of the OpenSim model data.

**FIGURE 3:**
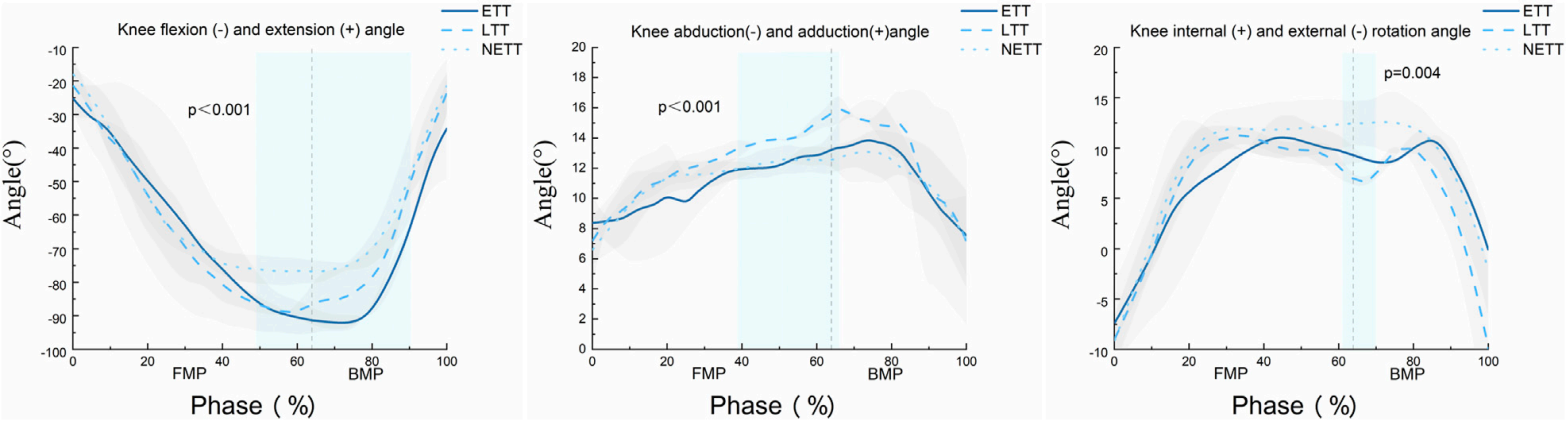
SPM1d analysis of the knee joint angle change curves for NETT, LTT, and ETT during the forward step bow stance movement in Tai Chi. The vertical dashed lines in the figure represent the moment of maximum anterior knee displacement during the bow step. The blue rectangular area indicates statistically significant differences among the three groups of movements within this region. The data are derived from the SPM1d{F} results.

As shown in Figure 3, the SPM1d{F} statistical results indicate that the position of knee joint forward movement significantly affects the knee flexion angle from 49% to 91% of the entire movement cycle (α = 0.05, F = 8.727, *p* < 0.01). During the 38%–67% phase of the movement, the position of knee joint forward movement significantly influences the knee adduction angle (α = 0.05, F = 11.02, *p* < 0.01). Additionally, in the 61%–70% phase of the movement, the position of knee joint forward movement significantly affects the knee internal rotation angle (α = 0.05, F = 10.385, *p* < 0.01).

**FIGURE 4:**
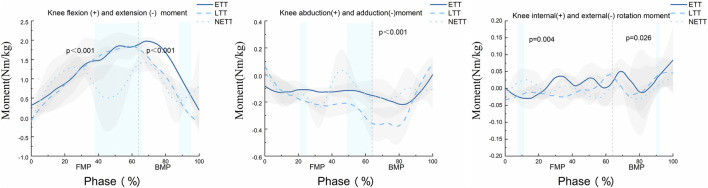
SPM1d analysis of the knee joint moment change curves for NETT, LTT, and ETT positions during the forward step bow stance movement in Tai Chi. The vertical dashed lines in the figure represent the moment of maximum anterior knee displacement during the bow step. The blue rectangular area indicates statistically significant differences among the three groups of movements within this region. The data are derived from the SPM1d{F} results.

The SPM1d{F} statistical results shown in Figure 4 indicate that the position of knee joint forward movement significantly affects knee flexion torque from 51% to 85% and 89%–95% of the entire movement cycle (α = 0.05, F = 10.795, *p* < 0.001). During the 21%–25% and 49%–63% phases of the movement, the position of knee joint forward movement significantly influences knee adduction torque (α = 0.05, F = 11.639, *p* < 0.001). Moreover, in the 8%–11% and 90%–91% phases of the movement, the position of knee joint forward movement significantly affects knee internal rotation torque (α = 0.05, F = 12.472, *p* < 0.001).

Table 2 shows significant correlations between anterior knee displacement position and various biomechanical outcomes. Specifically, the vertical ground reaction force in the ETT and LTT positions was significantly higher than in the NETT (*p* = 0.006). The knee anterior-posterior translation in the ETT and LTT positions was significantly higher than in the NETT (*p* < 0.001). This trend was also observed in the knee flexion angle, with ETT and LTT showing significantly larger angles than NETT (*p* = 0.001). Conversely, the knee internal rotation angle was significantly reduced in ETT and LTT compared to NETT (*p* = 0.029). Similarly, knee flexion torque in ETT and LTT was significantly higher than in NETT (*p* = 0.001), highlighting the impact of anterior knee displacement. Finally, this displacement also significantly increased stress on the anterior cruciate ligament (ACL), with ETT and LTT showing significantly higher stress levels than NETT (*p* = 0.011).

**TABLE 2:** Knee joint indicator parameters at NETT, LTT, and ETT positions during the forward step bow stance movement in tai chi (Mean ± SD).

Measure	NETT	LTT	ETT	F	p	η2
vGRF(N/BW)	1.25 ± 0.06^bc^	1.37 ± 0.03^a^	1.39 ± 0.02^a^	11.295	0.006	0.763
Knee anterior-posterior translation (mm)	0.39 ± 1.53^bc^	7.04 ± 0.83^a^	8.94 ± 0.56^a^	36.07	<0.001	0.927
Knee angle l (DEG)	−75.95 ± 4.19^bc^	−90.34 ± 1.48^a^	−92.61 ± 1.92^a^	27.445	0.001	0.887
Knee adduction l (DEG)	12.39 ± 0.87	14.89 ± 0.65	13.88 ± 1.58	4.204	0.063	0.546
Knee rotation l (DEG)	12.61 ± 2.16^bc^	8.77 ± 2.01^a^	8.53 ± 0.07^a^	6.125	0.029	0.636
Knee angle l moment (Nm/kg)	1.30 ± 0.18^bc^	1.84 ± 0.07^a^	1.98 ± 0.08^a^	22.232	0.001	0.864
Knee adduction l moment (Nm/kg)	−0.13 ± 0.03	−0.25 ± 0.03	−0.13 ± 0.11	2.808	0.127	0.445
Knee rotation l moment (Nm/kg)	−0.01 ± 0.03	−0.02 ± 0.002	0.04 ± 0.001	4.754	0.05	0.576
L ACL (N/BW)	0.89 ± 0.17^bc^	3.59 ± 0.35^a^	3.92 ± 1.08^a^	9.055	0.011	0.721

Table Note: 1. Significance comparisons between different groups are denoted by letters a-c.

^a^ Indicates a significant difference compared to the NETT, group.

^b^ Indicates a significant difference compared to the LTT, group.

^c^ Indicates a significant difference compared to the ETT, group.

2. The units for joint moment are Nm/kg, standardized based on the subjects’ body weight. Knee Moment and ground force are all standardized in N/BW (body weight) units.

3. The values defining the effect size indicator η2 are as follows: 0.01 represents a small effect, 0.06 represents a moderate effect, and 0.14 represents a large effect. The “Measure” data in the table represents the values of the left knee joint at the NETT, LTT, and ETT, positions during the forward lunge movement in Tai Chi.

**FIGURE 5:**
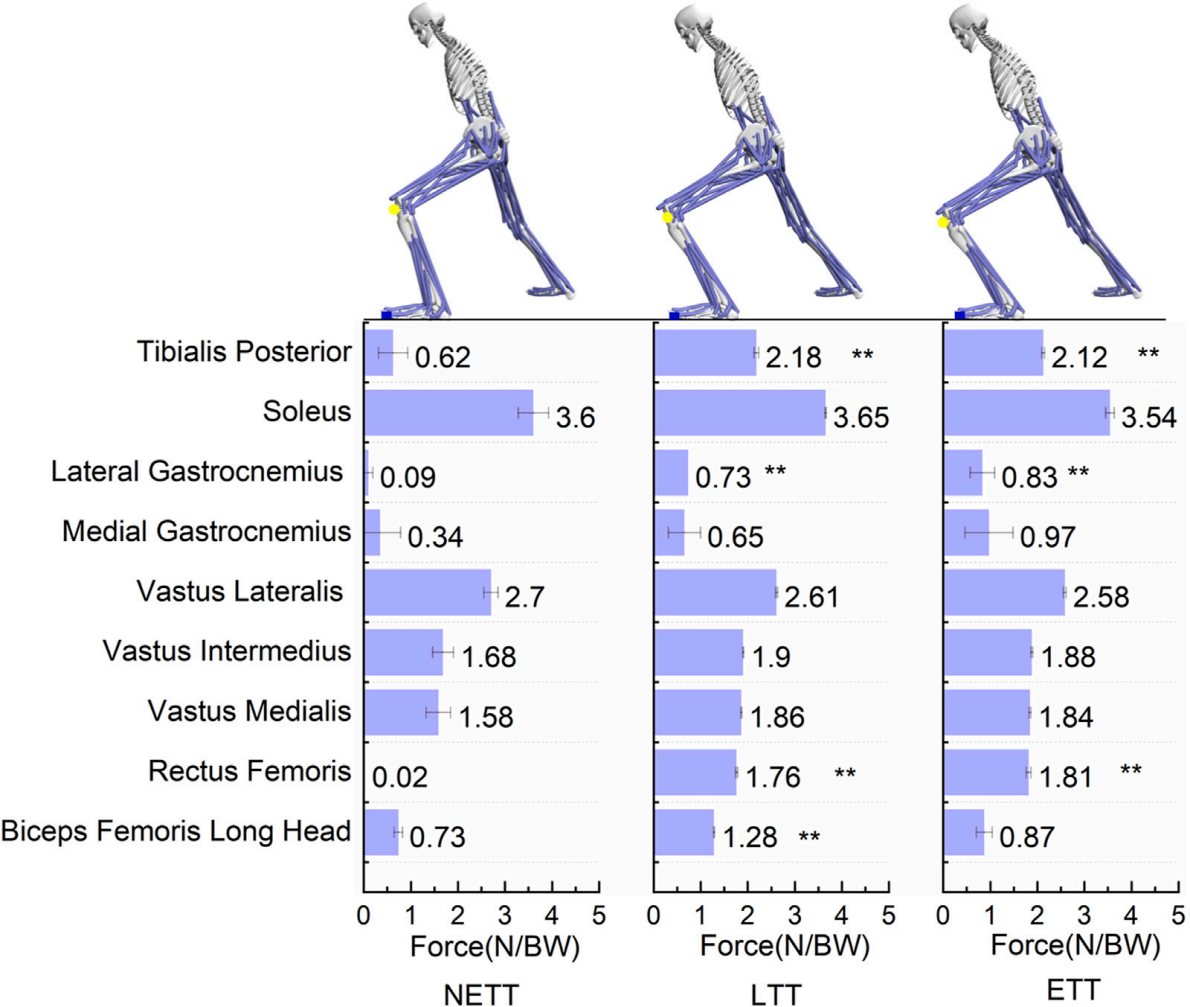
Comparison of muscle force differences around the knee joint at NETT, LTT, and ETT positions during the forward step bow stance movement in Tai Chi. **Indicates a significant difference from the NEET group at P<0.01. The force data of the lower limb muscles in the figure represents the values of the left knee joint at the NETT, LTT, and ETT positions during the forward lunge movement in Tai Chi.

As shown in Figure 5, the anterior movement of the knee at three different locations significantly affects the lower limb muscles. The tibialis posterior muscle force was significantly higher in FFT and ETT than in NETT (F = 7.637, *p* = 0.017). The strength of the vastus lateralis was significantly higher in FFT and ETT than in NETT (F = 24.933, *p* = 0.001). The degree of stress on the rectus femoris muscle in FFT and ETT was significantly higher than in NETT (F = 62.9, *p* < 0.001). The force of the long head of the biceps femoris in FFT was significantly higher than in NETT (F = 18.631, *p* = 0.002). The effect on the other muscles was relatively small (*p* > 0.05).

## 4 Discussion

This study investigates the impact of anterior knee joint displacement during the forward step bow stance movement in TC on knee joint load. The findings indicate that at specific anterior displacement positions, namely, LTT and ETT, the knee joint’s flexion-extension angle, flexion-extension torque, anterior cruciate ligament (ACL) loading, and surrounding muscle strength significantly increase compared to NETT. Notably, while previous research used simulation techniques to quantify the load on the lower limb joints during the forward bow step, our study provides new insights based on the existing literature.

The impact of vertical ground reaction force on knee joint load has been extensively studied. This study’s results indicate that vertical ground reaction forces at ETT and LTT are significantly higher than at NETT. During the heel-strike phase of TC, stress waves transmitted by ground reaction forces can exert substantial pressure on the lower limbs, potentially leading to knee joint injuries or gait alterations (Harithasan and Abd Razak, 2023). Despite the ground reaction forces in TC’s basic footwork is significantly lower than normal walking (Li et al., 2023), excessive anterior displacement of the knee joint increase vGRF and thus increase knee joint load, which is consistent with the hypothesis of the study.

Our results align with previous studies (Wen et al., 2018; Zhu et al., 2021), The spm1d statistical results in Figure 4 show that during the forward step phase of the TC bow stance, the flexion-extension torque at the NETT position is significantly lower than at LTT and ETT. This indicates that the primary impact of anterior knee joint displacement occurs during the forward step phase. After heel contacts with the ground, the position of forward movement of the knee joint increases, resulting in increased knee flexion. Since knee joint torque output is closely related to the flexion angle during anterior knee displacement (Kellis and Blazevich, 2022), when the anterior displacement of the knee joint exceeds the position of the toes, the force arm between the force point and the joint axis increases accordingly. Muscles must overcome greater torque to maintain or change the current angle. Even if the muscles apply the same force, the generated torque will be higher. Therefore, when the knee surpasses the toes, the movement of the body’s center of gravity increases the force arm acting on the knee joint flexion-extension torque (an increase of 19.2% in flexion torque at ETT compared to NETT). Moreover, our study revealed that the knee anterior-posterior translation in the ETT and LTT positions is significantly greater than that in the NETT position (*p* < 0.001). This finding highlights the potential challenges to knee stability in these specific positions. The marked increase in knee anterior-posterior translation may be associated with the biomechanical changes occurring in the knee joint during these positions, particularly in the forward translation phase. In the ETT and LTT positions, the anterior-posterior stability of the knee joint may be substantially influenced by the traction exerted by the tibia, which could lead to joint instability during movement and elevate the risk of injury. This explains why it is more difficult to keep the knee stable when the knee joint moves forward beyond the tip of the foot.

In the forward step bow stance of TC, a noteworthy observation is that the flexion angle of the knee joint continues to increase despite the knee reaching its predetermined position, as shown in Figure 3. This phenomenon possibly because the forward bow step requires relaxion, the center of gravity of the body moves forward and slightly downward and the position of the ribs relative to the ground is lowered. As a result, Knee flexion needs to be increased to adapt the shift of the center of gravity and maintain movement balance. The descent of the femur primarily enhances knee joint stability by improving the coordination and activity of the leg muscles, thereby supporting the knee joint and reducing the risk of injury (Liu et al., 2023). Another study suggests that during the movement, the descent and rotation of the femur help maintain balance and precision (Duan et al., 2020). This indicates that the forward step bow stance demands high coordination between the femur and pelvis as well as complex lower limb muscle activity. These muscle activity patterns help to strengthen lower limb strength and control. Therefore, in the TC bow stance, the descent of the femur is closely related to knee joint stability and muscle activity. Thus, it is speculated that during the forward lunge movement in Tai Chi, the anterior knee displacement may be a key kinematic factor affecting knee load and could serve as a relatively effective evaluation method. However, the reliability of this as a primary conclusion still requires further research for validation.

Previous studies have indicated that the knee adduction moment (KAM) is a key mechanical parameter for assessing knee joint loading (Gerus et al., 2013). However, there is no significant difference in the statistical results of knee adduction-abduction angles for three different knee forward positions. This consistency in KAM during the forward bow step may be attributed to the experimental design, which required participants to adopt a habitual gait, and the projection angle of the knee joint movement direction and the reference line is zero on the horizontal plane. Nonetheless, Figure 4 reveals that during the movement phase before reaching the predetermined knee forward position, the KAM at LTT was significantly higher than at ETT and NETT. This increase in KAM probably because the lunge posture requires the knee joint to align vertically with the toes, therefore, greater muscle strength is required to maintain stability, especially in the medial-lateral direction. This increased need for stability may lead to more tension in the inside muscle, such as the medial quadriceps, thereby increasing the KAM (Booij et al., 2020). This hypothesis is further supported by Figure 5, which shows that the muscle force of the vastus medialis in LTT is higher than in ETT. The study also suggests that the medial knee valgus moment may not accurately reflect the actual load on the medial compartment of the knee joint. The study conducted by Wang et al. employs a finite element musculoskeletal model (FEMS) to analyze the biomechanical changes in the knee joint resulting from radial tears of the medial meniscus during walking (Wang et al., 2022b). The findings indicate that larger radial tears of the medial meniscus may lead to increased stress concentration, which adversely affects the stability and health of the knee joint in Tai Chi. Therefore, it is essential to focus on the positioning and movement patterns of the knee joint to mitigate potential injury risks (Wang et al., 2022c). Future research should incorporate finite element musculoskeletal modeling techniques to comprehensively evaluate the load on the medial compartment and medial meniscus (Wen et al., 2018).

Previous studies have indicated a notable angle of internal rotation of the knee in TC’s bow stance movements (Wang C. et al., 2022). The results of this study show that the three types of TC stepping bow stances exhibit a significant angle of internal rotation of the knee, but there is no significantly statistical difference in internal rotation torque. Additionally, the internal rotation angle of the knee in the NETT stance is greater than in the LTT and ETT stances, likely due to optimizing mechanical leverage and muscle activation by positioning the knee behind the toes. This positioning achieves stable and effective movement, and this may lead to a larger internal rotation angle without compromising joint safety or efficiency (Zhu et al., 2021).

The relationship between knee forward movement and internal rotation angle involves complex interactions within the knee ligament structures, particularly the anterior cruciate ligament (ACL), which significantly impacts knee joint stability and mechanical function. In cases of insufficient ACL function, forward knee movement is usually accompanied by increased tibial internal rotation due to the lack of ACL’s stabilizing effect in such movements (Yim et al., 2015; Huser et al., 2017). This is consistent with the findings of this study, showing that in the NETT stance, the contraction force of the ACL is significantly lower than in the ETT and LTT stances, yet the internal rotation angle is higher. This suggests that under the NETT condition, the weakened function of the ACL effect leads to an increased internal rotation angle. As the forward movement of the knee increases, the body’s center of gravity shifts forward, and the shear force between the tibia and femur rises. This increase subsequently enhances the contraction force of the ACL to control the knee’s internal rotation and forward movement. Based on the results of this study, we found that both ACL tension and knee anterior-posterior translation increased with the forward displacement of the knee joint, which is consistent with previous research findings (Leppanen et al., 2021; Ruedl et al., 2023). The effort of the ACL and the muscles around the knee significantly affect the load changes on the knee joint. The ACL controls the knee’s forward movement, while the quadriceps also stabilize and control the knee joint (Maniar et al., 2018). As shown in Figure 5, the muscle force characteristics of lunges are similar in three different knee forward positions. The quadriceps primarily control the knee’s concentric and eccentric contractions during the bow stance’s forward movement, assisting joint stability and load distribution (Aslan et al., 2020). When the forward movement of the knee exceeds the tip of the toe in the bow stance, the triceps muscles, acting as the antagonistic muscles of the ACL, increases the load on the ACL, affecting the stability and mechanical load of the ACL (Sharifi and Shirazi-Adl, 2021). The results show that the force applied by the rectus femoris, the long head of the biceps femoris, the lateral gastrocnemius, and the tibialis posterior is significantly greater in ETT and LTT than in NETT. At small knee flexion angles, the rectus femoris’ force efficiency is low due to its smaller moment arm, generating less forward tibial load (Cavalcante et al., 2021; Garnier et al., 2021). In ETT and LTT, compared to NETT, the movement posture and amplitude of the femur will be significantly enlarged, thus increasing the force of the rectus femoris muscle. The lateral gastrocnemius maintains balance and stability by controlling knee flexion, directly affecting the ACL load, and plays an essential role in adjusting the knee joint’s dynamic stability (Schaarup et al., 2021; Raphaël Hamard et al., 2023). The tibialis posterior primarily helps control foot movement and stability in the bow stance, adjusting the soleus’s flexion to maintain foot stability, which is crucial for midfoot and hindfoot stability (Peng et al., 2021).Therefore, as the knee migration increases, muscle contraction force also rises, indicating that ETT exacerbates factors leading to strength imbalances in the knee joint, thereby increasing the knee joint’s load.

This study rigorously examines the impact of the anterior position of the knee joint during the advancing bow stance in Tai Chi on the load experienced by the knee joint. However, it has several limitations. First, the research participants did not include female athletes; therefore, future studies should include female subjects to explore the influence of gender factors on the experimental results. Second, future research should incorporate more comprehensive techniques and methodologies to further investigate the load on the medial compartment and medial meniscus of the knee joint.

## 5 Conclusion

This study compared the effects of three different knee forward positions on knee joint angles, torques, the anterior cruciate ligament, and surrounding muscles. The mechanical indicators of the knee in NETT were significantly lower than those in LTT and ETT. Additionally, it was confirmed that positioning the knee either behind or beyond the toes significantly increases the load on the knee joint during forward movement. The differences in knee load among the three conditions primarily occurred during the forward movement phase of the stepping bow stance.

## Data Availability

The original contributions presented in the study are included in the article/supplementary material, further inquiries can be directed to the corresponding authors.
